# Emotionally-oriented design in museums: a case study of the Jewish Museum Berlin

**DOI:** 10.3389/fpsyg.2024.1423466

**Published:** 2024-07-05

**Authors:** Zhihui Zhang, Jing Lu, Xiuying Zhang

**Affiliations:** ^1^Escola Tècnica Superior d'Arquitectura de Barcelona, Universitat Politècnica de Catalunya, Barcelona, Spain; ^2^Faculty of Architecture and City Planning, Kunming University of Science and Technology, Kunming, Yunnan, China; ^3^The Chinese University of Hong Kong, Hong Kong, Hong Kong SAR, China

**Keywords:** emotionally-oriented design, emotional architecture, museum design, architectural psychology, visitor experience, environmental psychology

## Abstract

**Objective:**

This study examines the intricate interplay between architectural design and visitor emotional responses at the Jewish Museum Berlin, focusing on how specific spatial elements such as the Holocaust Tower, Garden of Exile, The Voids, and The Axis elicit varied affective experiences. The research aims to extend the discourse on environmental psychology and architectural empathy, particularly within the context of memorial museums.

**Method:**

Employing a non-intrusive approach, the study gathered emotional response data using the Positive and Negative Affect Schedule (PANAS) from 113 museum visitors, with 102 valid responses analyzed. Environmental conditions such as light, sound, and spatial design were quantitatively measured to correlate with emotional responses captured at the end of visitors' tours across the designated museum spaces.

**Results:**

Findings revealed that architectural elements significantly influence emotional responses. High levels of negative emotions like fear and anxiety were markedly evident in the Holocaust Tower due to its minimal lighting and stark concrete structure. Conversely, the Garden of Exile induced more positive emotions through its use of natural light and greenery, emphasizing the role of biophilic design in enhancing emotional well-being. Statistical analysis supported these observations, with variations in emotional impact across different spaces demonstrating the profound effect of architectural design on visitor experiences.

**Conclusion:**

This study confirms that a variety of design elements and spatial strategies not only facilitate the presentation of historical narratives but also actively sculpt the emotional involvement and experiences of visitors. Our findings highlight the efficacy of emotionally-oriented architectural design in deepening the impact and engagement of museum visitors, emphasizing the transformative power of these environments in shaping visitor perceptions and interactions.

## 1 Introduction

Emotions, as complex psychological states involving subjective experiences, physiological responses, and behavioral expressions, are central to the human experience. They play a critical role in how we perceive, interact with, and remember our environments. In architectural contexts, emotions can range from awe and tranquility to anxiety and discomfort, influenced by elements such as spatial proportions, lighting, acoustics, and material finishes (Bower et al., 2019; Li, 2019; Shemesh et al., 2021; Zhang et al., 2022, 2023b). Understanding these emotional responses is crucial for creating spaces that not only serve functional needs but also foster well-being and meaningful experiences.

The interplay between architectural design and human emotion is a profound and complex subject that sits at the heart of environmental psychology and design studies. Architecture, transcending its utilitarian functions, wields the power to evoke a spectrum of emotions, shape behaviors, and create lasting memories. It is a tangible expression of culture and history, one that communicates and influences at a visceral level. Theoretical explorations in this domain affirm that the manipulation of light, volume, texture, and materiality in built environments can significantly sway individuals' mood states and psychological well-being (Webb, 2006; Ergan et al., 2018; Jafarian et al., 2018; Jiang et al., 2021; Zhang et al., 2022; Kim and Hong, 2023).

Consider the poignant role of memorial museums, where architectural design is tasked with the delicate balance of embodying historical narratives and facilitating reflective experiences. The Jewish Museum Berlin, designed by Daniel Libeskind, serves as a prime exemplar of how spatial design is intricately woven with emotional narrative (Sodaro, 2013; Tzortzi, 2017). This institution houses a confluence of spaces–The Axis, Garden of Exile, Holocaust Tower, and The Voids–each architecturally orchestrated to invoke distinct emotional responses from its visitors. The design embodies a dialogue between the stark realities of history and the potential for hope and renewal, leveraging the emotive capacity of architectural cues to guide visitors through a journey of collective memory and individual introspection (Feldman and Peleikis, 2014).

Given this context, the Jewish Museum Berlin was selected as a case study due to Libeskind's unique architectural vision. His design deliberately creates spaces intended to evoke both positive and negative emotions, which represents a significant departure from conventional architectural goals that typically prioritize comfort and positivity. This approach provides an ideal context for investigating how architectural elements shape emotional responses. By challenging visitors to engage with historical narratives on a deeply emotional level, Libeskind's design makes the Jewish Museum Berlin a compelling subject for studying the impact of architectural empathy.

The Axis, a metaphorical intersection of pathways, not only directs physical movement but also choreographs the emotional pacing of the visitor experience. The Garden of Exile, with its forest of pillars and disorienting angles, contrasts against the Holocaust Tower's imposing walls and constrained slivers of light, illustrating how light manipulation can be a powerful affective tool (Edensor, 2017; Zhang et al., 2022). The Voids, silent and resonant, offer a multisensory engagement that is as much about the presence of sound as it is about the voids of silence, echoing research that highlights the deep connection between sensory environments and emotional states (Henshaw and Mould, 2013; Fiebig et al., 2020; Algargoosh et al., 2022).

To effectively measure these emotional responses, various psychometric tools have been developed. One such tool is the Profile of Mood States (POMS), which assesses transient, distinct mood states through a series of adjectives rated by the respondent (McNair et al., 1971). Another is the Positive and Negative Affect Schedule (PANAS), which evaluates positive and negative affective states and is widely recognized for its reliability and validity in diverse settings (Watson et al., 1988). Additionally, tools like the Self-Assessment Manikin (SAM) provide a non-verbal pictorial assessment of emotional response, particularly useful in environments where verbal articulation may be challenging (Bradley and Lang, 1994).

Within this architectural milieu, the present study seeks to quantitatively investigate the emotional impact of these spaces on visitors. It draws upon the Positive and Negative Affect Schedule (PANAS) scale, a widely recognized metric for assessing affective dimensions (Watson et al., 1988). By grounding the subjective in the empirical, this research aims to contribute substantively to the dialogue on the empathetic capacity of architectural environments. It posits that designed spaces, particularly within the context of memorial museums, can function as catalysts for empathy, eliciting a range of emotions from contemplative sorrow to uplifting tranquillity (Watson, 2015; Golańska, 2015; Oren et al., 2022).

In synthesizing the PANAS findings with theoretical discourse, the study will explore how architectural form and content can act synergistically to enhance visitor engagement. It will address the interplay of memory, emotion, and place, offering insights into how spatial narratives can be thoughtfully constructed to resonate with visitors on an emotional and cognitive level. Such insights are anticipated to extend the current frameworks for architectural and environmental psychology, providing nuanced understandings of how spaces can be crafted to not just house experiences, but to actively shape and define them (Manzo, 2003; Shin, 2016).

Through this exploration, the study underscores the dynamic role of architecture in emotional storytelling within museum contexts. It is poised to offer valuable implications for design practices that seek to engage visitors beyond the visual, delving into the affective realm where architecture meets emotion, memory, and meaning (Lukas, 2012; Tolia-Kelly et al., 2017).

## 2 Method

### 2.1 Participants

This study adopted a non-intrusive method of data collection with visitors at the Jewish Museum Berlin, ensuring the authenticity of the emotional responses (Webb et al., 1999). Data were gathered from museum-goers at the conclusion of their visit to the Holocaust Tower, Garden of Exile, the “The Voids”, and the Axis spaces designed to provoke a range of emotional experiences. Research staff approached visitors at the exit of the museum, inviting those who had completed their tour to participate in the study. This strategy prioritized capturing the spontaneous emotional reactions of visitors, rather than pre-selected volunteers, thereby preserving the natural behavior and experiences within the museum environment.

Upon exiting, participants were asked to complete the Positive and Negative Affect Schedule (PANAS), a questionnaire that assesses a spectrum of emotional states triggered by the architectural and environmental attributes of the spaces visited. In addition to the PANAS, demographic data such as age, gender, and nationality were collected to facilitate a demographic breakdown of the emotional responses. The study engaged a total of 113 participants. After processing, 102 questionnaires were considered valid for analysis, with 11 discarded due to incompleteness. The demographics of the sample were diverse, with 59.46% identifying as female and 40.54% as male. The age distribution was as follows: 18–30 years old (33 participants), 31–40 years old (32 participants), 41–50 years old (31 participants), and over 50 years old (17 participants). All participants provided informed consent, ensuring ethical research practice.

### 2.2 Architectural space description

**Figure 1:**
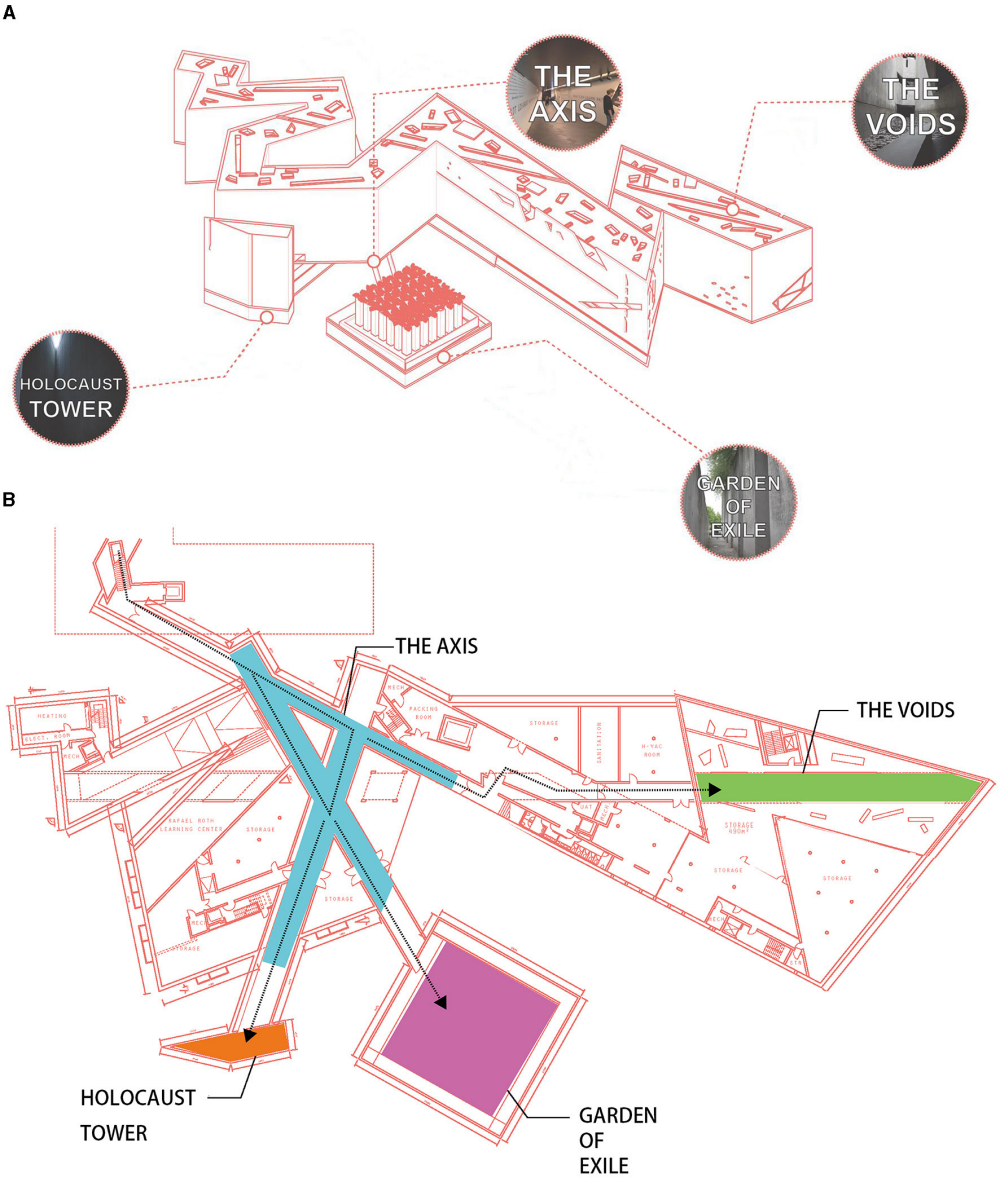
**(A)** Schematic Overview of the Jewish Museum Berlin: the four main architectural spaces explored in this study–Holocaust Tower, Garden of Exile, The Axis, and The “Voids.”, **(B)** Plan of the Jewish Museum Berlin.

The Jewish Museum Berlin, designed by Daniel Libeskind, is a striking example of contemporary memorial architecture. The museum comprises several distinctive spaces, each with its unique architectural features intended to evoke a range of emotional responses (Figure 1). Below is a comprehensive description of these spaces, encompassing their spatial dimensions and design solutions.

The Axis is a series of intersecting corridors that connect different parts of the museum. These pathways are designed to create a sense of direction and movement, guiding visitors through the museum's narrative. The walls are adorned with exhibits that provide context to the historical events commemorated by the museum. The Garden of Exile is an outdoor space featuring 49 concrete stelae, each 6 meters high, arranged in a grid pattern. The ground is tilted, creating a sense of disorientation and confusion, symbolizing the experience of exile. The stelae are filled with earth from Berlin and Jerusalem, emphasizing the connection between the past and the present. The Holocaust Tower is a tall, narrow, and empty space, measuring 24 meters in height with a small slit at the top allowing minimal natural light. The space is designed to evoke feelings of isolation, confinement, and introspection. The concrete walls and the stark, cold atmosphere contribute to the somber experience intended by the architect. The Voids are a series of empty spaces that run vertically through the building. These voids are intended to represent the absence of Jews in Berlin following the Holocaust. The largest of these voids, the Memory Void, contains an installation called “Shalekhet” (Fallen Leaves) by artist Menashe Kadishman, consisting of thousands of metal faces spread across the floor.

Libeskind's design employs sharp angles, irregular forms, and voids to convey the complexity and trauma of Jewish history. The use of materials such as concrete and steel, along with the interplay of light and shadow, enhances the emotional impact of the spaces. These architectural solutions are not merely aesthetic but are deeply symbolic, intended to engage visitors on both an intellectual and emotional level.

### 2.3 Measures

The Positive and Negative Affect Schedule (PANAS) was used to assess participants' emotional responses. PANAS is a widely recognized scale that measures two dimensions of affect: Positive Affect (PA) and Negative Affect (NA). Each dimension consists of 10 items. Participants rate the extent to which they feel each emotion on a scale from 1 (very slightly or not at all) to 5 (extremely). The Positive Affect items include interested, excited, strong, enthusiastic, proud, alert, inspired, determined, attentive, and active. The Negative Affect items include distressed, upset, guilty, scared, hostile, irritable, ashamed, nervous, jittery, and afraid (Watson et al., 1988).

### 2.4 Environmental measurements

**Figure 2:**
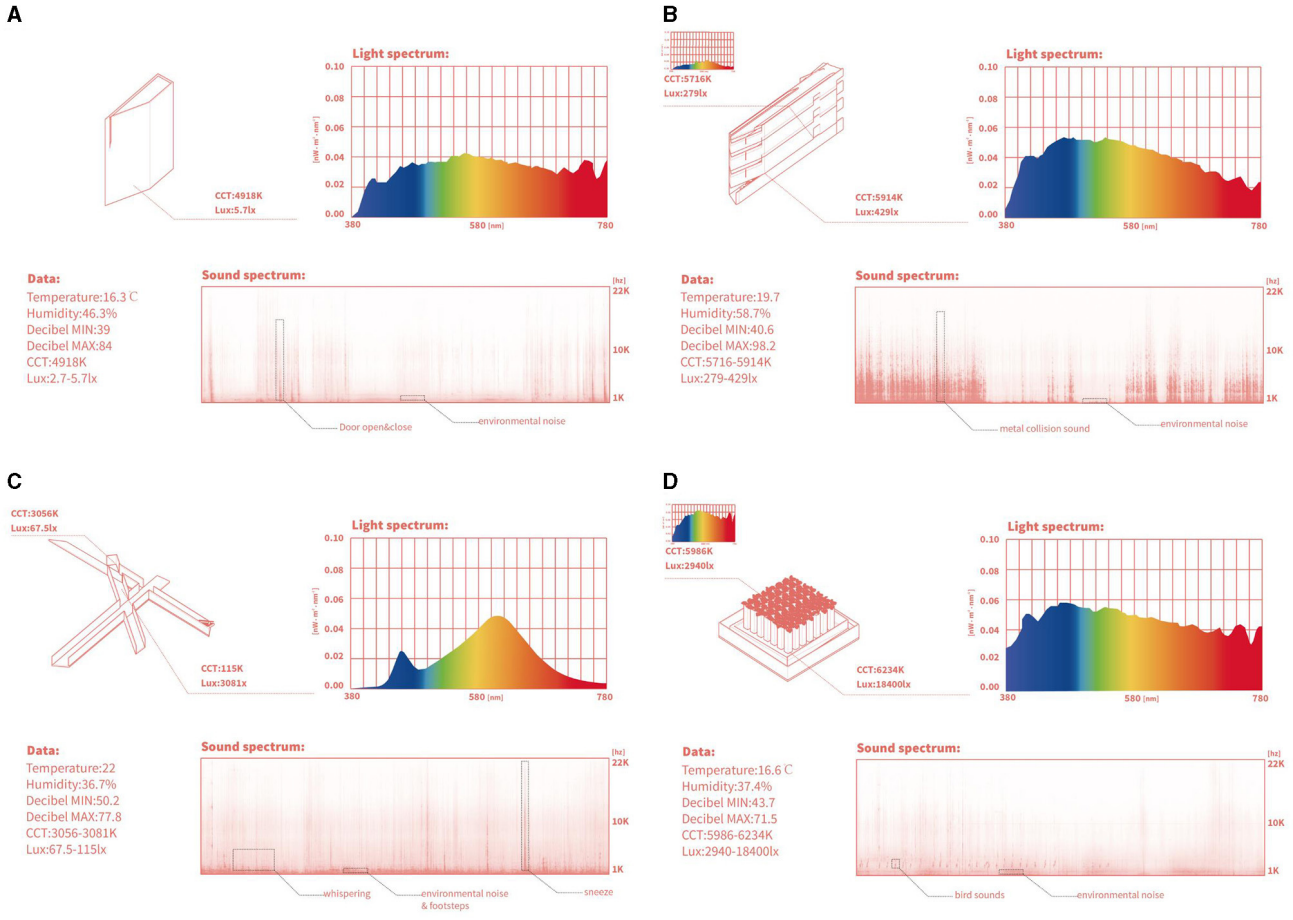
Comparative Environmental Measurements across Four Spaces of the Jewish Museum Berlin: **(A)** Holocaust Tower, **(B)** The Voids, **(C)** The Axis, and **(D)** Garden of Exile. Each panel presents light and sound spectra, accompanied by temperature, humidity, and decibel range data.

In the Jewish Museum Berlin, comprehensive environmental and acoustic measurements were gathered from four distinct spaces using a suite of instruments: a sound level meter (Smart sensor AS804), light meter (UNI-T UT383), temperature and humidity meter (UNI-T UT333), audio recorder (Tascam DR-100MK III), and spectrometer (Sekonic C-700). These instruments were employed to collect precise data, as shown in Figure 2.

The purpose of presenting the data about color temperature, light intensity, sound levels, temperature, and humidity is to provide a detailed environmental context for each space within the museum. These environmental factors are known to significantly influence human emotional and psychological responses (De Rojas and Camarero, 2008). By measuring and documenting these parameters, the study aims to explore how specific environmental conditions may influence the emotional responses reported by visitors, thereby offering a more comprehensive understanding of how architectural elements affect visitor experiences (Goulding, 2000; Bigné et al., 2005; Halpenny, 2010).

For example, in the Holocaust Tower, illumination ranged from 2.7 to 5.7 Lux, with a natural spectrum color temperature of 4918K. The low lighting and cooler color temperature contribute to the overall somber and reflective atmosphere of the space. The temperature was recorded at 16.3 °C, and humidity at 46.3%, with sound levels varying between 39 and 84 decibels. The significant height of the space, 21 meters, created an echo effect, intensifying the auditory experience and potentially heightening feelings of isolation and introspection.

The Garden of Exile was illuminated much more variably, between 2,940 and 18,400 Lux, with natural light color temperatures from 5986 to 6234K, temperature at 16.6 °C, and humidity at 37.4%. Sound levels here ranged from 43.7 to 71.5 decibels, with ambient sounds such as bird calls enriching the outdoor environment. The variation in light intensity and the presence of natural elements like vegetation are intended to evoke feelings of confusion, displacement, but also a sense of connection to nature, promoting reflection and contemplation.

In “The Voids”, indoor natural light levels were measured from 279 to 429 Lux and color temperatures between 5716 to 5914K. The space was warmer at 19.7 °C and more humid at 58.7%, with sound levels reaching up to 98.2 decibels due to the presence of art installations and specific architectural acoustics. These environmental characteristics contribute to a complex sensory experience that engages visitors on multiple levels.

Finally, The Axis, defined as a pathway, recorded illumination levels from 67.5 to 115 Lux and cooler color temperatures of 3056 to 3081K. The temperature there was around 22 °C and humidity at 36.7%. Sound levels, affected by visitor interactions, reached up to 77.8 decibels, with LED lighting that limited the depth of sensory engagement.

The time frame used for measuring the sound spectrum in each space was standardized to a continuous 10-minute interval during peak visiting hours. This period was selected to capture the typical ambient noise levels and visitor interactions within each environment. The audio recordings were analyzed to determine the average and peak decibel levels, as well as the frequency distribution of sounds, ensuring a comprehensive acoustic profile of each space.

By presenting these environmental measurements, the study aims to explore how the physical characteristics of each space may influence the emotional reactions they elicit. This approach provides a nuanced understanding of how specific environmental conditions might contribute to the overall emotional impact of architectural design in a museum setting.

### 2.5 Analysis strategy

The study utilized the Positive and Negative Affect Schedule (PANAS) to measure emotional responses after visitors explored four distinct sections of the Berlin Jewish Museum: the Holocaust Tower, The Axis, The Voids, and the Garden of Exile. Data processing was meticulously carried out using Python, leveraging libraries such as Pandas for data manipulation and NumPy for numerical operations. This ensured the precision of the PANAS scores, which are crucial for assessing the immediate impact of each spatial design on visitors' emotions (Hovy, 2022).

Statistical analyses were conducted using independent samples t-tests to evaluate emotional variances across different spatial elements, with 'The Axis' serving as a baseline comparison. This analysis was facilitated by the SciPy library, a tool integral to executing statistical tests in Python. Additionally, the effect sizes were computed using Cohen's d, providing insights into the magnitude of emotional responses elicited by each architectural element (Howell, 1992).

The Axis was selected as the baseline for comparison due to its transitional nature and relative neutrality in emotional design. Unlike the Holocaust Tower, The Voids, and the Garden of Exile, which are explicitly designed to evoke strong emotional responses, The Axis serves primarily as a connective pathway linking different parts of the museum. Additionally, The Axis features exhibits along its sides, embodying characteristics typical of a standard museum exhibition space. This makes it an ideal reference point for measuring variations in emotional impact across more emotionally charged spaces. By using The Axis as a baseline, the study can more accurately isolate and identify the specific emotional influences of the other architectural elements.

The entire analysis was conducted and documented using a Jupyter Notebook, which facilitates the integration of live code with narrative text, enhancing the clarity and reproducibility of the research. We adhered to a conventional significance threshold of *p* < 0.05 throughout our analyses to ensure the statistical validity of our findings. The use of the SciPy library was central to our statistical analysis, allowing us to perform robust t-tests and calculate effect sizes efficiently. This, along with other Python tools such as Pandas for data manipulation, greatly streamlined the process and enhanced our ability to manipulate and visualize data effectively (Virtanen et al., 2020; McKinney, 2022).

By employing robust statistical tools and a reliable data analysis environment, the study effectively quantified the emotional impacts of architectural design, setting a precedent for future research in the domain of emotional architecture.

## 3 Result

### 3.1 Emotional responses across different spaces

This study conducted a comprehensive analysis of emotional responses to the architectural spaces within the Berlin Jewish Museum, namely the Holocaust Tower, The Axis, The Voids, and the Garden of Exile. Data extracted from Table 1, which displays the average PANAS (Positive and Negative Affect Schedule) scores for each space, reveals nuanced patterns in emotional engagement.

**Table 1:** Average PANAS scores for emotional responses across architectural spaces at the Jewish Museum Berlin: the table delineates the intensity of each emotion reported by visitors in the Holocaust Tower, The Axis, The Voids, and the Garden of Exile.

	**Holocaust tower**	**The axis**	**The voids**	**Garden of exile**
Interested	2.25	2.36	3.99	4.06
Distressed	3.75	2.46	3.12	1.61
Excited	2.08	2.62	2.82	3.66
Upset	3.44	2.72	3.86	1.70
Strong	2.26	2.18	2.80	3.49
Guilty	1.97	1.70	1.93	1.38
Scared	3.71	2.89	3.07	1.68
Hostile	2.99	2.54	2.71	1.61
Enthusiastic	1.83	2.07	2.07	3.71
Proud	1.80	2.26	1.73	3.17
Irritable	2.75	2.31	2.49	1.56
Alert	4.13	2.99	3.72	2.44
Ashamed	1.57	1.54	1.43	1.45
Inspired	1.99	2.47	2.33	3.59
Nervous	3.94	3.25	3.29	1.91
Determined	2.63	2.93	3.09	3.95
Attentive	2.98	2.73	3.95	3.96
Jittery	3.68	2.53	3.36	1.71
Active	2.12	2.64	2.67	4.02
Afraid	3.80	2.10	3.33	1.56

Scores are based on a scale where 1 indicates “very slightly or not at all,” 3 signifies “moderately” or “somewhat,” and 5 denotes “extremely”.

**Low affect emotions:** Emotions such as guilt (Guilty) and shame (Ashamed) consistently scored low across all spaces, with average scores of 1.97, 1.70, 1.93, and 1.38 for Guilty and 1.57, 1.54, 1.43, and 1.45 for Ashamed in the Holocaust Tower, The Axis, The Voids, and Garden of Exile respectively. These low scores suggest that the museum's exhibits are less likely to evoke feelings of personal responsibility or embarrassment, likely reflecting the thematic elements focused more on historical reflection than on personal culpability.

**Stable affect emotions:** Emotions such as alertness (Alert) and activeness (Active) manifested moderately across all spaces, indicating a general state of engagement. For example, Alert scores were 4.13, 2.99, 3.72, and 2.44, while Active scores were 2.12, 2.64, 2.67, and 4.02 across the Holocaust Tower, The Axis, The Voids, and Garden of Exile respectively. These scores indicate that the museum's design consistently engages visitors, maintaining their attention and physical activity throughout the exhibits.

**Highly variable emotions:** Within the Berlin Jewish Museum, thematic and design differences had a pronounced impact on emotional responses in specific spaces. The Holocaust Tower exhibit, for instance, significantly elicited higher levels of distress-related emotions, with fear (Afraid) and anxiety (Nervous) scoring 3.80 and 3.94 respectively. These heightened scores likely reflect the intense historical context that the exhibit aims to convey. In stark contrast, the Garden of Exile area proved to be a space that fostered positive emotional states, achieving scores of 3.59 for inspiration (Inspired) and 4.06 for interest (Interested). These results highlight the Garden of Exile's effectiveness in evoking feelings of reflection and positive engagement, showcasing how different environmental themes can distinctly influence visitor emotions.

**Positive emotions:**

**Figure 3:**
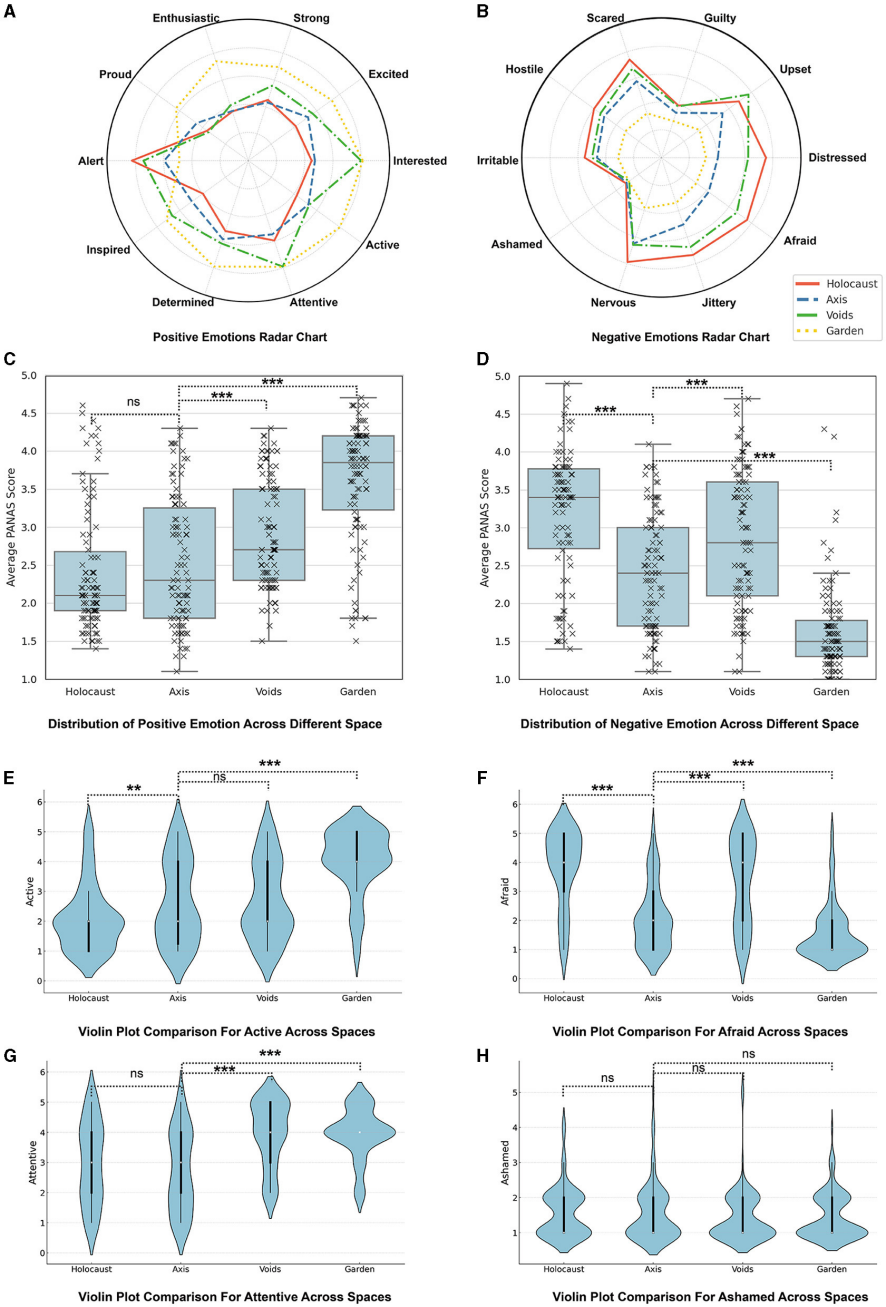
Emotional profiles and distributions in the Jewish Museum Berlin's architectural spaces: **(A)** Positive emotions radar chart. **(B)** Negative emotions radar chart. **(C)** Box plot of the distribution of positive emotion PANAS scores across different spaces. **(D)** Box plot of the distribution of negative emotion PANAS scores across different spaces. **(E)** Violin plot comparison for active across spaces. **(F)** Violin plot comparison for afraid across spaces. **(G)** Violin plot comparison for attentive across spaces. **(H)** Violin plot comparison for ashamed across spaces. Asterisks denote significance levels (^*^p < 0.05, ^**^p < 0.01, ^***^p < 0.001).

Active (Figure 3E): Among the positive emotions, the feeling of activeness showed the largest variance across the spaces. The scores for Active were 2.12 in the Holocaust Tower, 2.64 in The Axis, 2.67 in The Voids, and significantly higher at 4.02 in the Garden of Exile. This results in a maximum difference of 1.90, indicating that the Garden of Exile notably enhances visitors' feelings of activeness compared to the other spaces.

Attentive (Figure 3G): In contrast, the emotion of attentiveness exhibited the smallest variance among the positive emotions. Scores were 2.98 in the Holocaust Tower, 2.73 in The Axis, 3.95 in The Voids, and 3.96 in the Garden of Exile, with a maximum difference of 1.23. This suggests a relatively consistent level of attentiveness across all exhibits, with slightly higher engagement in the latter two spaces.

**Negative emotions:**

Afraid (Figure 3F): For negative emotions, the feeling of fear (Afraid) showed the greatest variance. Scores were 3.80 in the Holocaust Tower, 2.10 in The Axis, 3.33 in The Voids, and 1.56 in the Garden of Exile, resulting in a maximum difference of 2.24. This indicates that the Holocaust Tower significantly elicits higher levels of fear compared to the other spaces, reflecting its intense historical context.

Ashamed (Figure 3H): Conversely, the emotion of shame (Ashamed) exhibited the smallest variance among negative emotions. The scores were 1.57 in the Holocaust Tower, 1.54 in The Axis, 1.43 in The Voids, and 1.45 in the Garden of Exile, with a minimal maximum difference of 0.14. These consistently low scores suggest that the museum's exhibits are less likely to evoke feelings of personal responsibility or embarrassment, likely due to their focus on historical reflection rather than personal culpability.

### 3.2 T-test results of emotional responses

Drawing on data visualized in the radar charts (Figures 3A, B) and the box plots (Figures 3C, D), this study offers an in-depth examination of the emotional responses to different spatial environments within the Berlin Jewish Museum: Holocaust Tower, The Axis, The Voids, and Garden of Exile. The Positive and Negative Affect Schedule (PANAS) scores provide a nuanced exploration of how each space influences visitors' emotional states.

From the radar charts, it's evident that certain spaces amplify specific emotions. The Holocaust Tower exhibit, as shown in Figure 3B, registers pronounced distress-related responses, particularly fear and anxiety, while the Garden of Exile, depicted in Figure 3A, facilitates positive emotions such as inspiration and interest.

The box plots in Figures 3C, D quantify these observations:

- **Holocaust Tower vs. The Axis:** While the Holocaust Tower space did not significantly differ from the Axis in terms of positive emotional responses (t-statistic of -0.997; p-value of 0.320; Cohen's d of -0.14), it markedly elevated negative emotions (t-statistic of 6.625; *p*-value of 3.15 × 10^−10^; Cohen's d of 0.93), as Figure 3D illustrates.
- **The Voids vs. The Axis:** Figure 3C reveals that The Voids space engendered a significant increase in positive emotions compared to the Axis (t-statistic of 3.555; *p*-value of 4.74 × 10^−4^; Cohen's d of 0.50). Figure 3D reflects a similar trend in negative emotions (t-statistic of 3.849; p-value of 1.60 × 10^−4^; Cohen's d of 0.54).
- **Garden of Exile vs. The Axis:** As the most positively impactful environment, the Garden of Exile's influence on positive emotions is statistically significant (t-statistic of 9.376; *p*-value of 1.48 × 10^−17^; Cohen's d of 1.31). Conversely, it substantially reduces negative emotions (t-statistic of -8.174; *p*-value of 4.24 × 10^−14^; Cohen's d of -1.14), reinforcing its restorative role as seen in Figure 3D.

These detailed results, grounded in statistical analysis and visual evidence, underscore the profound and varied emotional impacts that architectural elements have on museum visitors, highlighting the importance of thoughtful spatial design in influencing visitor experience.

## 4 Discussion

The findings of this study provide a comprehensive analysis of the influence of architectural design on emotional responses, as illustrated by the experiences of visitors to the Jewish Museum Berlin. The statistical data from the PANAS questionnaires, along with the radar charts (Figures 3A, B) and box plots (Figures 3C, D), provided a multidimensional view of how each distinct space within the museum evoked varying emotional states among the participants.

### 4.1 Interpretation of findings

The results of this study, particularly the heightened negative emotional responses in the Holocaust Tower exhibit, suggest a complex interplay of environmental factors rather than solely the impact of low lighting on mood. Contrary to the expected calming effects of low lighting identified in studies by de Ruyter and van Dantzig (De Ruyter and Van Dantzig, 2019), Kombeiz (Kombeiz et al., 2017), and others, the Holocaust Tower's unique combination of minimal natural light, the stark, cold concrete architecture, and the towering voids, uniquely contributed to visitors' feelings of fear and anxiety (Campens, 2017). This distinct atmosphere, characterized by its chilling austerity and vast, empty spaces, was effectively aligned with the exhibit's thematic intent to invoke deep reflection on a dark period in history.

Conversely, the Garden of Exile leverages the principles of biophilic design through its integration of natural light and vegetation, echoing Evensen et al.'s assertion of nature's positive effects on human emotion (Dash, 2017; Evensen et al., 2017). This space consistently evoked feelings of inspiration and interest among visitors, suggesting that biophilic elements in architectural design can significantly contribute to the promotion of positive emotional states.

Furthermore, the auditory experience provided by “The Voids” sound installations played a crucial role in shaping the museum's emotional atmosphere, substantiating DeNora's findings on the emotive power of sound (DeNora, 2000; Ebbensgaard, 2017; Tavakoli et al., 2017). This multisensory approach appears to have been successful in enhancing visitors' emotional engagement, underpinning the significance of considering auditory elements within architectural spaces.

This study extends the current discourse on environmental psychology by empirically demonstrating the differential emotional impacts elicited by distinct architectural elements within a museum context. The substantial variance in emotional responses to each space underlines the potential for architecture to serve not merely as a backdrop for exhibits but as an active participant in the storytelling process of a museum. These insights offer valuable contributions to the field, suggesting that architectural design, when thoughtfully executed, has the power to evoke a deeply emotional narrative and profoundly affect the visitor experience.

### 4.2 Implications for architectural design

The quantitative findings of this research underscore the complex role that architectural spaces play as dynamic mediators of emotional experience within museums. The design of the Axis in the Jewish Museum Berlin, acting as a connector between the various thematic spaces, effectively sets the stage for an emotional transition, thereby heightening the contrast in affective responses as visitors move from one space to another.

This relational dynamic between the spaces suggests that the emotional impact of a museum visit is not solely dependent on the artefacts displayed but is significantly influenced by the journey the architecture curates. The Axis, therefore, serves a pivotal role in the emotional narrative of the museum, underpinning the importance of considering the sequence of spatial experiences in museum design. It is an interesting and essential aspect that architects and designers need to deliberate upon–the emotional interplay between successive spaces and its cumulative effect on visitor engagement.

The empirical evidence from this study supports the argument for a holistic approach to museum architecture, one that includes the intentional use of varying architectural elements to evoke and modulate emotions throughout the visitor's journey. The affective dimension of spatial design, as observed in the Jewish Museum Berlin, is a testament to the capacity of thoughtful architectural planning to not only showcase exhibits but also to elicit a spectrum of emotions that enrich the overall narrative and experience.

### 4.3 Limitations and future research

The present study, while offering valuable insights, has several limitations that warrant mention. The timing of the questionnaires was not synchronized with the measurement of environmental factors, which may affect the accuracy of capturing visitors' immediate emotional responses, particularly in the Garden of Exile space. As an outdoor area, the Garden is subject to environmental and weather variations, making it challenging to ensure a consistent experience for all visitors. This variability was not controlled for in the study and represents a potential confound in interpreting the emotional impact of this space.

The discrepancy in timing between the visitors' experiences and the administration of the PANAS questionnaire could lead to recall bias, where participants may not accurately remember or may reinterpret their emotional states after the fact. Cultural backgrounds and the age of participants were also not factored into the analysis, which could influence the interpretation of the emotional responses elicited by the museum's spaces. Additionally, the possibility of repeat visits by participants was not a consideration in the study's design, potentially affecting the novelty of the experience and subsequent emotional responses.

To address these limitations, future research could consider employing virtual reality (VR) technology to simulate the museum environment under controlled conditions, ensuring uniformity in visitors' experiences regardless of external factors such as weather. VR technology allows for the control of environmental variables, providing a consistent and replicable experience for all participants. Additionally, VR can facilitate the collection of physiological measures of emotional responses, such as galvanic skin response or heart rate variability, providing a more objective and nuanced understanding of the emotional effects of architectural spaces. Moreover, VR technology can be used to capture and analyze facial expressions to quantify emotions, adding another layer of emotional data. This method, as demonstrated in previous research (Zhang et al., 2023a), can provide real-time insights into participants' emotional states, offering a more comprehensive assessment of their experiences. Further investigation with a larger and more diverse sample, taking into account cultural and age differences, would also be beneficial in enhancing the generalizability of the findings to other commemorative architectural contexts. This approach would ensure that the emotional impacts of architectural design are understood across a broad spectrum of visitors, contributing to more inclusive and effective design strategies.

## 5 Conclusions

This study tentatively suggests that architectural elements such as lighting, vegetation, and sound may have a significant impact on the emotional responses of visitors to the specific spaces analyzed within the Jewish Museum Berlin. The findings indicate that minimal lighting in the Holocaust Tower likely intensified visitor experiences of sombre reflection, while the use of natural light and greenery in the Garden of Exile might have enhanced feelings of inspiration and interest. Similarly, sound installations in The Voids appear to have deepened the emotional engagement of the visitors.

These preliminary observations propose that the thoughtful integration of architectural and environmental factors can potentially enrich the visitor experience in commemorative spaces, offering a nuanced approach to museum design that goes beyond traditional exhibit presentation. However, it is important to note that this study focused solely on four specific spaces within the museum and did not include the exhibition spaces. Therefore, while our findings provide valuable insights, further research is necessary to confirm these observations and to explore their applicability in other settings within memorial museums.

## Data availability statement

The original contributions presented in the study are included in the article/supplementary material, further inquiries can be directed to the corresponding author.

## Ethics statement

The studies involving humans were approved by Universitat Politécnica de Catalunya Ethics Committee. The studies were conducted in accordance with the local legislation and institutional requirements. The participants provided their written informed consent to participate in this study.

## Author contributions

ZZ: Conceptualization, Data curation, Formal analysis, Investigation, Methodology, Validation, Visualization, Writing – original draft, Writing – review & editing. JL: Funding acquisition, Methodology, Resources, Validation, Visualization, Writing – original draft, Writing – review & editing. XZ: Conceptualization, Funding acquisition, Investigation, Methodology, Visualization, Writing – original draft, Writing – review & editing.
